# Hereditäres Angioödem durch C1-Inhibitor-Mangel, eine nationale Versorgungsleitlinie

**DOI:** 10.1007/s00508-023-02298-x

**Published:** 2023-12-08

**Authors:** Werner Aberer, Sabine Altrichter, Urban Cerpes, Thomas Hawranek, Clemens Schöffl, Tamar Kinaciyan

**Affiliations:** 1 Universitätsklinik für Dermatologie und Venerologie, Medizinische Universität Graz, 8036 Auenbruggerplatz 8, Graz, Österreich; 2 Universitätsklinik für Dermatologie und Venerologie, Kepler Uniklinikum, Linz, Österreich; 3 Universitätsklinik für Dermatologie und Allergologie, Uniklinikum Salzburg, Salzburg, Österreich; 4 Universitätsklinik für Dermatologie, Medizinische Universität Wien, Wien, Österreich

**Keywords:** Quincke-Ödem, Schwellung, Langzeitprophylaxe, Seltene Erkrankung, Lebensqualität, Quinke edema, Swelling, Longterm prophylaxis, Orphan disease, Quality of life

## Abstract

Das hereditäre Angioödem (HAE) ist eine seltene, schmerzhafte, behindernde und potenziell tödliche Erkrankung, bei der eine frühzeitige Diagnose und wirksame Therapie von entscheidender Bedeutung sind. Die vorliegende österreichische Leitlinie zur Diagnose und Behandlung von HAE bietet Anleitungen und Informationen zum State-of-the-Art-Management von HAE speziell in Österreich, und dies im Gegensatz zu globalen Richtlinien, in denen die spezifische Situation aller Länder weltweit berücksichtigt werden muss. Unser Ziel ist es, österreichischen Ärzten dabei zu helfen, HAE als Differenzialdiagnose bei entsprechenden Symptomen zu erkennen und zu berücksichtigen, rationale Entscheidungen für die Diagnose und Behandlung von HAE mit C1-Inhibitor-Mangel (Typ 1 oder Typ 2) zu treffen, indem wir hier über häufige und wichtige klinische Symptome, Diagnosemethoden, Behandlungsmodalitäten wie verfügbare HAE-spezifische Medikamente in Österreich informieren, und nicht zuletzt, um sie zu motivieren, ihre Patient:innen zur Bestätigung der Diagnose und zur adäquaten Behandlungsentscheidung in HAE-Zentren vorzustellen.

## Abstract (en)

Hereditary angioedema (HAE) is a rare, painful, disabling and potentially fatal disease, where early diagnosis and effective treatment are critical. These Austrian guidelines for the diagnosis and management of HAE provide instructions and advice on the state of the art management of HAE in Austria in contrast to global guidelines, where the situation of all countries worldwide must be taken into account. Our goal is to help Austrian physicians to consider HAE as a differential diagnosis with corresponding symptoms, to make rational decisions for the diagnosis and management of HAE with C1-inhibitor deficiency (type 1 or type 2). The guidelines provide information on common and important clinical symptoms, diagnostic methods, treatment modalities, available HAE-specific medications in Austria and last but not least to motivate physicians to refer patients to HAE centers for confirmation of the diagnosis and adequate treatment decisions.

## I. Ziel der Leitlinie

Die Leitlinie beruht auf dem informellen Konsens von Experten, die sich in Österreich seit vielen Jahren mit dem Krankheitsbild hereditäres Angioödem (HAE) aufgrund eines genetischen C1-Inhibitor(C1-INH)-Mangels (HAE-C1-INH) befassen, und einer nichtsystematischen Literaturrecherche. Diese Leitlinie stellt eine Aktualisierung der deutschen AWMF-Leitlinie aus dem Jahr 2019 [1] und der internationalen WAO/EAACI-Leitlinie aus dem Jahr 2021 [2] dar. Hauptzweck ist die Adaptierung der in diesen Leitlinien ausgesprochenen Empfehlungen auf die spezifische Situation von Patient:innen und deren Betreuer:innen in Österreich. Dies ist wichtig, weil sich für österreichische Patient:innen in der Diagnostik und noch viel mehr in der Therapie die Möglichkeiten deutlich von den internationalen Empfehlungen unterscheiden – und dies zugunsten der österreichischen Patient:innen. In einer rezenten Studie konnte nämlich gezeigt werden, dass Zulassung und Verfügbarkeit der neuen Therapeutika in den verschiedenen Ländern und Regionen der Erde sehr unterschiedlich sind und dies weitgehend mit dem Bruttosozialprodukt korreliert [3]. Internationale Leitlinien wie die der WAO/EAACI [2] müssen aber berücksichtigen, dass Betroffenen in vielen Ländern die modernen Präparate nicht zur Verfügung stehen, sondern nur die „alten“ Substanzen mit schlechtem Nutzen-Nebenwirkungs-Profil verfügbar sind – die aber in Österreich nicht mehr eingesetzt werden sollten.

Die Leitlinie richtet sich in erster Linie an Ärzt:innen, die Patient:innen mit HAE-C1-INH behandeln. Dies sind in Österreich meist Dermatologen; und dies aufgrund der häufigsten Manifestationsform, dem kutanen Angioödem. Die Leitlinie soll aber auch Ärzt:innen ansprechen, die potenziell Kontakt mit HAE-C1-INH haben; dies sind vorwiegend Allgemeinmediziner:innen, Gastroenterolog:innen, HNO- und Zahnärzt:innen und Pädiater:innen, aber auch Anästhesist:innen und Notfallmediziner:innen, an die sich Patient:innen mit dieser seltenen Krankheit mit ihren Symptomen, meist Hautschwellungen, wenden. Die zielführende Diagnostik erfolgt oft zeitlich verzögert, sodass viele Betroffene einen ungewöhnlich langen Leidensweg erleben: Die Zeit von der Erstmanifestation bis zur Diagnose betrug in Österreich bis 2017 durchschnittlich mehr als 20 Jahre [4]. Und etliche Betroffene werden noch immer mit Medikamenten behandelt, die heute aufgrund ihres ungünstigen Nebenwirkungsprofils bei uns in Österreich obsolet sind, da neue, gut-verträgliche Therapeutika ein weitgehend „normales“ Leben ermöglichen könnten.

Der internationalen Empfehlung [2] folgend, sollte heute jede:r HAE-Patient:in einen individualisierten Aktions- und Behandlungsplan und eine HAE-spezifische, allumfassende – „comprehensive“ – ganzheitliche Betreuung erhalten. Und weil dies bei dieser seltenen Erkrankung – „orphan disease“ – nicht einfach ist, sollte die Betreuung jedes dieser Patient:innen durch eine:n Spezialist:in mit spezifischer Expertise im Management von HAE erfolgen.

### Empfehlung 1

Wir empfehlen, dass eine HAE-spezifische umfassende, integrierte Versorgung für alle Patient:innen verfügbar ist. Diese sollte von ein:er Spezialist:in mit besonderer Erfahrung in der Behandlung von HAE koordiniert werden.

## II. Begründung für diese nationale Leitlinie

Neue wissenschaftliche und klinische Erkenntnisse, begleitet von innovativen therapeutischen Optionen für den Notfall und für die Prophylaxe, haben in der vergangenen Dekade zu signifikanten Änderungen in den Betreuungsempfehlungen für HAE geführt. Leitlinien für den nationalen Gebrauch [1] ebenso wie spezielle für Kinder/Jugendliche [5, 6] sowie Frauen/Schwangere/Stillende [7] bezeugen dies eindrucksvoll. Darauf aufbauend hat eine internationale Leitlinie mit Experten aus allen Erdteilen versucht, globale Standards für das Management von HAE-Patient:innen zu etablieren [2]. Mittels der vorliegenden Leitlinie sollen diese Standards auf die österreichische Situation für Betroffene übertragen werden, da sich diese von der internationalen sowohl in der Verfügbarkeit diagnostischer Maßnahmen und noch viel mehr bei den therapeutischen Möglichkeiten deutlich unterscheidet. So sind die in der internationalen Leitlinie [2] als „second-line“ eingestuften Therapeutika in Österreich heute obsolet, da die First-line-Medikamente allesamt verfügbar und den Vorgängerpräparaten bezüglich Wirk‑/Nebenwirkungspotenzial deutlich überlegen sind: Ihr Wirkpotenzial ist gut belegt und das Nebenwirkungsprofil vorteilhaft.

### Empfehlung 2

Jeder Betroffene soll einen individuellen Aktionsplan haben, der regelmäßig aktualisiert werden muss.

## III. Allgemeines

**Figure 1:**
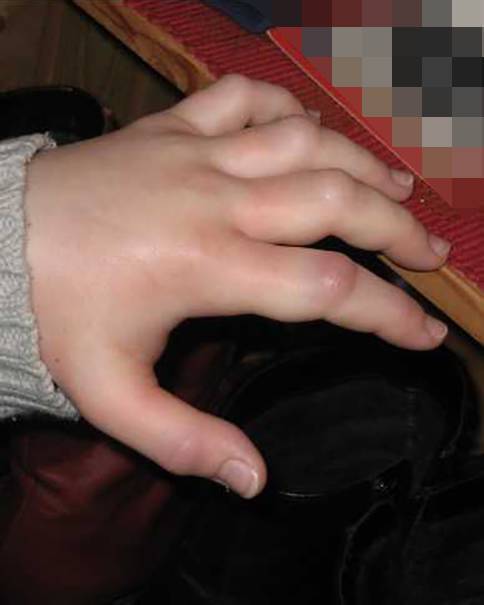

HAE ist eine seltene, potenziell lebensbedrohliche, erbliche Erkrankung, die sich durch rezidivierende Schwellungsepisoden im Bereich des subkutanen bzw. (sub-)mukosalen Gewebes manifestiert (Abb. 1). Die Schwellungsattacken treten unregelmäßig und unvorhersehbar auf, entwickeln sich über mehrere Stunden (12–36 h) bis zum Vollbild und dauern ca. 2 bis 5 Tage an, bevor sie sich wieder vollkommen rückbilden. Häufig treten Schwellungen an der Haut der Extremitäten (Hände, Füße, Arme, Beine), im Magen-Darm-Trakt, weniger häufig im Gesicht und im Urogenitaltrakt auf. Schwellungen im Bereich der Atem-Schluck-Straße treten selten auf, können jedoch aufgrund der beengten anatomischen Lokalisation bereits nach kurzer Zeit lebensbedrohlich werden. Todesfälle durch Ersticken wurden und werden leider auch weiterhin berichtet [8].

## IV. Epidemiologie

Untersuchungen in 6 europäischen Ländern ergaben, dass die Prävalenz von HAE, bedingt durch einen C1-INH-Mangel, stabil ist und etwa 1,5 Fälle pro 100.000 beträgt [9]. Die genaue Schätzung der Prävalenz wird jedoch durch die Seltenheit der Krankheit und den damit verbundenen geringen Bekanntheitsgrad, das Fehlen etablierter überregionaler Register, die begrenzte Forschung und unterschiedliche Methoden der Datenerhebung erschwert. Neuere Daten aus Österreich [10] zeigen regionale Unterschiede in der Prävalenz von HAE-C1-INH, wobei einige Regionen eine Prävalenz von etwa 1:30.000 aufweisen, während in anderen Bundesländern nur wenige oder gar keine HAE-C1-INH-Patient:innen bekannt sind. Diese Unterschiede beruhen aber wohl auf einer unterschiedlichen Detektions- bzw. Meldefrequenz, da bisher keine Unterschiede in verschiedenen Ethnien beschrieben wurden. Männer und Frauen sind gleich häufig betroffen.

## V. Pathogenese

Ursächlich für HAE-C1-INH ist ein autosomal-dominant vererbter Gendefekt im SERPING1-Gen, welches für den C1-INH codiert. Dieser Faktor ist ein zu den Serpinen gehörender Serin-Proteaseinhibitor, der die Aktivierung des Komplementfaktors C1 kontrolliert und so das Komplementsystem reguliert. Bis dato sind etwa 700 verschiedene Mutationen in besagtem Gen beschrieben [11]. Biochemisch lassen sich 2 Typen des HAE-C1-INH unterscheiden, wobei der Typ 1 mit 85 % häufiger vorkommt; bei diesem wird das defekte oder deletierte Gen nicht exprimiert, beim HAE-C1-INH-Typ 2 wird das C1-INH-Protein zwar hergestellt, es ist jedoch nicht funktionstüchtig. Laborchemisch zeigen sich bei beiden Formen erniedrigte C1-INH-Funktionswerte, quantitativ findet sich beim Typ 1 eine erniedrigte, beim Typ 2 eine normwertige bis erhöhte C1-INH-Konzentration. Das Protein wird überwiegend in Hepatozyten gebildet. Der C1-INH wirkt regulatorisch im Komplementsystem, bei der Fibrinolyse, im Koagulationssystem und im Kallikrein-Kinin-System, wobei sich ein Mangel nur in Letzterem phänotypisch mit rezidivierenden Haut- und Schleimhautschwellungen manifestiert. Grund dafür ist die nicht ausreichende Hemmung des Enzyms Kallikrein durch C1-INH und die somit gesteigerte Bradykininbildung. Bradykinin bindet an den Bradykinin-B2-Rezeptor am Endothel und führt lokal zur Vasodilatation und Permeabilitätssteigerung und damit zum Angioödem. Neben erniedrigten C1-INH-Werten finden sich zumeist erniedrigte Komplementfaktor-C4-Werte durch die mangelnde Inhibierung der ersten Schritte des Komplementsystems.

Weitere, sich phänotypisch ähnlich präsentierende und pathophysiologisch verwandte Angioödemformen, welche jedoch mit normwertigen C1-INH-Werten einhergehen, sind bekannt und werden unter HAE-nC1-INH geführt (s. Diagnostik) [12]. Deren Pathophysiologie ist zum Teil noch unklar, diese Leitlinie beschränkt sich daher weitgehend auf HAE-C1-INH.

## VI. Klinische Symptome

Betroffene erleben in variablen Abständen teils tagelange schmerzhafte Episoden mit heftigsten, kolikartigen, schneidenden Bauchschmerzen mit Erbrechen und Diarrhö im Rahmen von Bauchattacken. Schwellungen an den Extremitäten, im Genitalbereich und im Gesicht können schmerzhaft, massiv funktionseinschränkend und entstellend sein und im schlimmsten Fall, bei Affektion der Atem-Schluck-Straße, auch tödlich verlaufen [8].

Prodromalzeichen können, müssen aber nicht auftreten. An der Haut kann sich ein Erythema marginatum ausbilden (ein hellrotes, polyzyklisches, nicht juckendes, makulöses Exanthem), es können vor einer Schwellungsattacke auch prickelnde Sensationen auftreten. Müdigkeit und Reizbarkeit werden gelegentlich berichtet sowie Übelkeit, Völlegefühl oder ein Hungergefühl, welche Vorboten einer Bauchattacke sein können. Dysurische Beschwerden sind bei Schwellungen im Urogenitalbereich möglich. Wichtig sind die Prodromalzeichen bei einer sich anbahnenden Schwellungsattacke im Bereich der Atem-Schluck-Straße, hierbei kann es zur Stimmveränderung, Heiserkeit sowie zu Schluckbeschwerden kommen.

Angioödeme können an jeder Körperstelle entstehen, zumeist sind jedoch die Extremitäten und der Gastrointestinaltrakt betroffen. Bis zu 50 % der HAE-C1-INH-Patient:innen berichten anamnestisch von lebensbedrohlichen Halsschwellungen [8].

Die ersten Symptome treten meist im Kindes- und Jugendalter auf. Bis zur korrekten Diagnose vergehen aber oftmals mehrere Jahre. Bei den österreichischen Patient:innen dauert es im Mittel 15 Jahre (sofern keine Familienmitglieder mit einer nachgewiesenen HAE-Diagnose bekannt sind); auch bei „jüngeren“ HAE-Betroffenen (nach 1980 geboren) beträgt der durchschnittliche „diagnostic delay“ immer noch 7,5 Jahre [10].

## VII. Auslöser und Triggerfaktoren

Die meisten Schwellungsattacken treten ohne einen offensichtlichen Auslöser auf. Es müssen jedoch Medikamente gemieden werden, die Attacken auslösen können wie ACE-Hemmer oder östrogenhaltige Kontrazeptiva. Des Weiteren können Infekte, mechanische Traumata, operative Eingriffe vor allem im Kopf‑/Halsbereich und ungewohnte körperliche Belastungen Angioödemattacken triggern [13]. Für manche Betroffene stellen emotionale Erlebnisse sowie Stresssituationen den wichtigsten Auslöser dar. Eine personalisierte Beratung ist bei diesem Thema unumgänglich.

## VIII. Verlauf

Die Erstmanifestation erfolgt meist zu Beginn des zweiten Lebensjahrzehnts. Frequenz und Intensität der Attacken variieren zwischen den Patient:innen und korrelieren mit keinem der bekannten Krankheitsparameter. Frauen sind oft stärker betroffen. Bei Schwangeren können die Attacken häufiger oder auch seltener auftreten. Im höheren Lebensalter verlaufen die Krankheitsschübe bei manchen Patient:innen mit etwas abgeschwächter Symptomatik; selten sistieren die Ödemattacken vollkommen [1].

## IX. Diagnostik

Der Verdacht auf HAE sollte gestellt werden, wenn sich ein:e Patient:in mit wiederkehrenden Schwellungen der Haut (vor allem an Extremitäten, Gesicht und Genitalien), gastrointestinalen Attacken (schmerzhafte Bauchkrämpfe) und/oder Ödem im Kopf‑/Halsbereich vorstellt [1, 2]. Der Verdacht auf HAE wird weiter erhärtet, wenn ein Patient eines oder mehrere der folgenden Merkmale aufweist:

1. eine positive Familienanamnese (bei ca. 75 % der Betroffenen),
2. Beginn der Symptome in der Kindheit/Jugend,
3. wiederkehrende und schmerzhafte Bauch‑/Unterleibssymptome,
4. Auftreten einer Schwellung der oberen Atemwege (Zunge, Kehlkopf),
5. Nichtansprechen auf Antiallergika wie Antihistaminika, Glukokortikoide und Epinephrin oder den Anti-IgE-Antikörper Omalizumab,
6. Vorhandensein von Prodromalzeichen oder -symptomen (z. B. figurierte Erytheme),
7. das gleichzeitige Fehlen von Quaddeln.

### 1. Biochemische Diagnostik

Primär sollte eine biochemische Diagnostik (Laboratoriumsdiagnostik im Plasma) bei klinischem Verdacht auf ein HAE durchgeführt werden. Folgende Parameter sollen dabei bestimmt werden:

- C1-INH-Aktivität,
- C1-INH-Konzentration,
- C4-Konzentration.

Beweisend für einen C1-INH-Mangel sind Werte von weniger als 50 % der C1-INH-Aktivität und weniger als 50 % der C1-INH-Konzentration im Vergleich zu Normalwerten. In Einzelfällen kann C4 im Plasma normal sein (Tab. 1).

**Table 1:** 

	C1-INH-Aktivität	C1-INH-Proteinkonzentration	C4-Proteinkonzentration
HAE-Typ 1	↓	↓	↓
HAE-Typ 2	↓	Normal oder ↑	↓
Andere HAE-Typen	Normal	Normal	Normal oder ↓

Durch einen „Suchtest“ mit nur einem dieser Parameter lässt sich ein HAE-C1-INH weder beweisen noch ausschließen. Testergebnisse, die auf HAE-1/2 hinweisen, sollten bestätigt werden, d. h. die Tests sollten wiederholt werden, idealerweise in einem zertifizierten Labor. Das Gleiche gilt für inkonsistente Ergebnisse oder Ergebnisse, die im Widerspruch zum Phänotyp stehen. HAE hat zahlreiche und lebenslange Folgen für die Patienten und ihre Familien; daher muss die Diagnose auf bestätigten Testergebnissen beruhen.

Die Tests auf C1-INH sind störanfällig [14] und werden überdies von vielen Labors nur selten durchgeführt, was das Risiko falsch positiver und falsch negativer Ergebnisse birgt. Die Empfehlung, Tests auf C1-INH-Funktion, C1-INH-Protein und C4 bestätigen zu lassen, bezieht sich nur auf die Erstdiagnose von HAE. Es gibt keine Indikation für abermaliges Testen bei schon gesicherter Diagnose. Überdies ist zu beachten, dass die Bestätigung von HAE durch Wiederholungstests bei Patient:innen, die positiv getestet wurden, eine wirksame Behandlung nicht verzögern darf.

**Figure 2:**
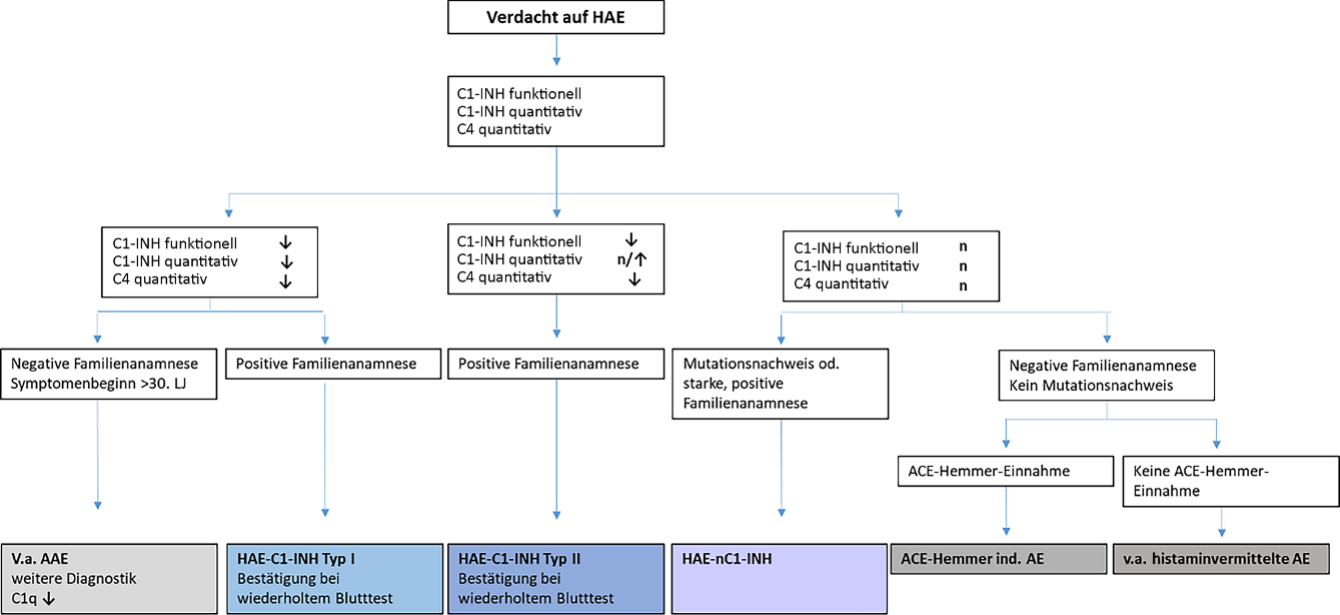

Die Diagnose HAE-C1-INH Typ 1 oder 2 kann gestellt werden bei (Abb. 2):

- rezidivierenden peripheren Schwellungen der Haut und/oder abdominellen Schmerzattacken und/oder Schwellungen im oberen Atemtrakt,
- den zugehörigen Laborbefunden,
- gegebenenfalls der positiven Familienanamnese (negativ bei Neumutation).

Eine Bestätigung mittels genetischer Untersuchung ist bei klarem Ergebnis nicht zwingend [1, 2].

#### Empfehlung 3

Bei jedem Verdacht auf HAE-C1-INH sollen laborchemisch C1-INH-Aktivität, C1-INH-Konzentration und C4 bestimmt werden.

#### Empfehlung 4

Bei pathologischen Werten muss eine Kontrolluntersuchung, vorzugsweise an einem Zentrum mit einem zertifizierten Labor, erfolgen.

### 2. Genetische Diagnostik

Bei biochemisch eindeutigen Befunden sind genetische Untersuchungen bei HAE-C1-INH unnötig. Die heute angewandten molekulargenetischen Untersuchungen haben eine Detektionsrate von 90–95 %, falsch negative Befunde sind also selten. Bei Patienten, bei denen trotz Kontrolle aufgrund z. B. widersprüchlicher Ergebnisse der Plasmauntersuchung keine exakte Diagnose möglich ist, oder bei Patienten mit positiver Familienanamnese ohne laborchemische Pathologie sind sie deshalb sinnvoll. Diese Patient:innen sollten sich in spezialisierten Behandlungszentren vorstellen und die Indikation für eine genetische Untersuchung dem Spezialzentrum überlassen werden. Eine Mutationssuche im *C1-INH*-Gen wird derzeit in einigen Genetiklaboren in Österreich angeboten (Tab. 2).

**Table 2:** 

	Genmutationen
*HAE Typ 1/2*	„Serin proteinase inhibitor G1“ (SERPING1)
*Publizierte HAE-nC1-INH-Typen*	Faktor XII (F12) Plasminogen (PLG) Angiopoietin 1 (ANGPT1) Kininogen 1 (KNG1) Myoferlin (MYOF) Heparan sulfate(HS)-glucosamine 3-O-sulfotransferase 6 (HS3ST6)

#### Empfehlung 5

Wir empfehlen, Patienten mit Verdacht auf HAE, die normale C1-INH-Spiegel und -Funktion aufweisen, auf Mutationen zu untersuchen, die für HAE-nC1-INH detektiert und publiziert worden sind.

### 3. Pränatale Diagnostik

Eine pränatale Diagnose durch genetische Untersuchung von Chorionzotten oder Amnionflüssigkeit ist möglich. Voraussetzung ist, dass eine ursächliche Mutation in der Familie mit HAE bereits bekannt ist. Eine solche Untersuchung sollte nur bei strenger Indikationsstellung durchgeführt werden und ist daher selten notwendig.

### 4. Postnatale Diagnostik bei Kindern

Aufgrund schwankender Komplementwerte im ersten Lebensjahr sind die oben genannten 3 diagnostischen Plasmaparameter frühestens nach Beendigung des ersten Lebensjahres verlässlich [16]. Trotz eingeschränkter Zuverlässigkeit kann eine Diagnostik vor dem Erreichen des ersten Lebensjahres aber Hinweise auf das Vorliegen eines HAE geben.

#### Empfehlung 6

Kinder aus HAE-betroffenen Familien sollen so früh wie möglich getestet werden.

### 5. Familienuntersuchungen

Die Untersuchung von Blutsverwandten auf die 3 genannten biochemischen Parameter ist erforderlich. Dies ist von besonderer Bedeutung, weil auf diese Weise schwere Schwellungsattacken mit Komplikationen, wie z. B. Erstickungsfälle bei Nicht-Diagnostizierten, vermieden werden können.

Neumutationen (s. oben) sind möglich und kommen in 25 % der Fälle vor, hierbei zeigen dann Eltern und Geschwister Normalbefunde bei den Plasmaparametern [1, 2].

#### Empfehlung 7

Bei nachgewiesenem C1-INH-Mangel sollen alle blutsverwandten Familienmitglieder auf einen C1-INH-Mangel untersucht werden.

### 6. Differenzialdiagnose

Die Differenzialdiagnose umfasst das erworbene Angioödem durch C1-INH-Mangel (AAE-C1-INH), die chronische Urtikaria (die mit Angioödemen einhergehen kann) sowie die durch ACE-Inhibitoren getriggerten Angioödeme ([1, 2]; Tab. 3).

**Table 3:** 

Differenzialdiagnose	Anamnese	Ursachen	Diagnostik
*Erworbenes Angioödem* *AAE-C1-INH*	Später Beginn (nach dem 30. LJ) Familienanamnese negativ	Lymphom, benigne monoklonale Gammopathie (MGUS)	C1-INH-Funktion, C1-INH-Protein und C4 ↓, C1q ↓ (bei 75 % der Patienten)
*ACE-Hemmer induzierte Angioödeme*	ACE-Hemmer Einnahme	Ev. HAE-Typ 1/2	Keine spezifische, C1-INH-Funktion, C1-INH-Protein und C4 zum Ausschluss eines HAE
*Mastzellvermittelte Angioödeme*	Häufig juckende Quaddeln	Mastzellaktivierung, Histamin-vermittelt	Keine spezifische, C1-INH-Funktion, C1-INH-Protein und C4 zum Ausschluss eines HAE

Symptome eines HAE-C1-INH können fehldiagnostiziert werden (z. B. Bauchattacken als Appendizitis), umgekehrt sind nicht alle Symptome immer auf das HAE zurückzuführen, und sämtliche Differenzialdiagnosen (insbesondere bei GI-Beschwerden) sollten auch bei HAE-Patienten abgeklärt werden.

AAE-C1-INH kommt seltener vor als HAE-Typ 1/2. Die Symptome sind denen von HAE-1/2 ähnlich, und das grundlegende diagnostische Laborprofil ist nicht von HAE-Typ 1/2 zu unterscheiden. Zu den Unterschieden gehören ein später Beginn der Symptomatik im höheren Lebensalter (nach dem 30. LJ), eine negative Familienanamnese, Grunderkrankungen wie ein Lymphom oder eine benigne monoklonale Gammopathie (MGUS), gelegentliche konstitutionelle Symptome (B-Symptomatik) und häufig erniedrigte C1q-Spiegel. C1q ist bei 75 % der Patient:innen mit AAE-C1-INH niedrig, kann aber auch normal sein, insbesondere bei Patienten, die anabole Androgene einnehmen. Viele Patient:innen mit AAE-C1-INH haben Autoantikörper, die C1-INH inaktivieren, welche jedoch nur in Speziallaboren bestimmt werden können.

Patienten, bei denen ACE-Hemmer-induzierte Angioödeme auftreten, sollten auf HAE-1/2 getestet werden, da das Auftreten von Angioödemattacken nach Beginn der Behandlung mit einem ACE-Hemmer auf ein zuvor asymptomatisches HAE hinweisen kann. Es liegt im Wirkmechanismus der ACE-Hemmer, dass der Bradykininspiegel steigt und sich in Folge Angioödeme ausbilden können.

Sehr häufig treten wiederkehrende Angioödeme bei Patient:innen mit chronisch spontaner Urtikaria (CSU) auf. Diese mastzellvermittelten Schwellungen sind häufig mit stark juckenden Quaddeln verbunden. Einige CSU-Patient:innen entwickeln jedoch keine Quaddeln und haben ausschließlich Angioödeme. Wichtig ist, dass CSU eine häufige Erkrankung ist, die auch HAE-Patient:innen betreffen kann. Das Auftreten von Quaddeln schließt daher nicht unbedingt ein HAE aus, und das Fehlen von Quaddeln schließt ein mastzellvermitteltes Angioödem nicht aus. Nicht sedierende Antihistaminika in Standard- oder höheren als den Standarddosen, allein oder in Kombination mit Omalizumab oder Immunmodulatoren wie Cyclosporin, können Quaddeln und Angioödeme bei CSU-Patient:innen verhindern. Ein Therapieansprechen mit diesen Medikamenten bestätigt diese Diagnose. Da mastzellvermittelte Angioödeme weitaus häufiger sind als HAE-1/2, ist ein primärer Therapieversuch mit Antihistaminika und, falls erforderlich, mit Kortikosteroiden und Epinephrin angezeigt, wenn die Diagnose noch nicht feststeht und die Anamnese nicht zwingend auf ein HAE hinweist.

## X. Therapie

### 1. Therapieziele

Therapieziele sind einerseits die rasche und effektive Behandlung aller akuten Attacken und dadurch Vermeidung eines Erstickungstodes bei Larynxattacken [8] oder Zungenschwellungen sowie Attacken-bedingter Schmerzen [17] und andererseits die Verminderung der Krankheitsaktivität (Häufigkeit, Schwere und Dauer der Attacken) durch Langzeitprophylaxe (LTP). Damit verbunden ist die Normalisierung des Lebens für die Betroffenen durch bessere Planbarkeit von schulischen, beruflichen und privaten Aktivitäten und Steigerung der Lebensqualität, die auch in Attacken-freien Phasen massiv beeinträchtigt ist durch die Sorge, dass jederzeit unerwartet eine schwere Attacke auftreten könnte [2].

Die LTP wird heute nicht nur von den Betroffenen oder Patientenorganisationen, sondern auch von den behandelnden Ärzten gefordert, die das Leid der Patient:innen hautnah miterleben. Dieser berechtigten Forderung kommt die Industrie zunehmend nach und investiert in die Entwicklung neuer Medikamente, die direkt in den Pathomechanismus des HAE eingreifen und bei guter Verträglichkeit Attacken effektiv verhindern.

#### Empfehlung 8

Wir empfehlen, dass die Ziele der Behandlung in der vollständigen Kontrolle der Krankheit und der Normalisierung des Lebens der Patient:innen bestehen.

### 2. Allgemeine Maßnahmen

Aufklärung der neu diagnostizierten Betroffenen und der Familienangehörigen über die schwere Symptomatik der Erkrankung wie massive schmerzhafte Schwellungen in allen Körperbereichen, gastrointestinale Beschwerden wie Koliken Durchfall, Erbrechen, Schwellung der Bauchdecke und nicht zuletzt über potenzielles Ersticken durch Larynxattacken. Zusätzlich werden Betroffene durch erfahrene HAE-Ärzte idealerweise in HAE-Zentren über potenzielle Triggerfaktoren informiert, die individuell relevanten Trigger ausgearbeitet sowie das Gespräch schriftlich dokumentiert.

Im Rahmen dieses Gespräches wird auch die individuelle Medikamentenanamnese erhoben und, falls Östrogen-haltige Präparate (orale Kontrazeptiva, Hormonersatztherapie, IUDs/Hormonspirale), ACE-Hemmer oder Sartane eingenommen werden, wird das Absetzen dieser Medikamente eingeleitet, da sie die Häufigkeit und Schwere der Attacken verursachen bzw. deutlich erhöhen.

Im Anschluss an das Gespräch wird ein Therapieplan erstellt, entsprechende Rezepte werden ausgestellt und Betroffene mit einem Notfallausweis ausgestattet [2].

### 3. Medikamentöse Therapie

Prinzipiell wird zwischen 2 therapeutischen Strategien unterschieden: i) sofortige Therapie beginnender Attacken aus Erfahrung der Patient:innen mit sogenannter Bedarfsmedikation, On-demand-Therapie. Diese soll idealerweise von den Betroffenen selbst applizierbar sein, sodass Attacken sich durch frühe Behandlung möglichst rasch wieder zurückbilden. Dies ist besonders wichtig bei Gesichts- oder Zungenschwellungen, weil sie öfters in Larynxattacken münden [8]. In solchen Fällen soll nach der akuten Behandlung auch ein HAE-Arzt oder ein Spital aufgesucht werden; ii) oder eine Dauertherapie insbesondere bei häufigen und schweren Attacken zur Verhinderung idealerweise aller, aber zumindest möglichst vieler Attacken. Die Dauertherapie wird in der Fachsprache auch LTP genannt. Da keine der zurzeit verschreibbaren LTP-Medikamente bei allen Betroffenen alle Attacken verhindern und es dennoch vereinzelt zu sogenannten Durchbruchsattacken kommen kann, muss allen LTP-Patient:innen auch On-demand-Therapie in einer Dosierung, die für die Behandlung von 2 Attacken ausreicht, verordnet werden.

#### Empfehlung 9

Wir empfehlen, dass allen HAE-Betroffenen, auch jenen mit LTP, immer eine On-demand-Therapie für mindestens 2 Attacken verordnet wird.

#### 3.1. Therapiemöglichkeiten für akute HAE-Attacken

##### 3.1.1. C1-INH-Konzentrat

Zwei Präparate stehen zur Verfügung, beide aus humanem Plasma gesunder Spender gewonnen, pasteurisiert, virusinaktiviert, nanofiltriert und gefriergetrocknet. Sie müssen kurz vor der intravenösen Injektion aufgelöst werden.

Das erste Präparat ist in Europa seit 1985 unter dem Namen Berinert® (CSL Behring, King of Prussia, PA, US) auf dem Markt. Es besteht langjährige Erfahrung über die gute Wirksamkeit bei allen Formen von akuten Attacken [1]. Die Zulassung in den USA erforderte eine randomisierte kontrollierte Studie (RCT-Studie) mit 125 HAE-Patient:innen, in der diverse Konzentrationen zum Einsatz kamen. 20 IE pro kg Körpergewicht (KG) ergab gegenüber Placebo eine statistisch signifikante Wirksamkeit, wobei innerhalb von 30 min erste Besserung unabhängig vom Schweregrad der Attacke eintrat [18].

Das zweite C1-INH-Präparat, Cinryze® (Takeda, Tokio, Japan) kam 2011 für die Behandlung akuter HAE-Attacken auf den europäischen Markt. Dieses sieht eine fixe Dosierung von 1000 IE vor [19]. Cinryze® ist für die akute Attackenbehandlung für Kinder ab 2 Jahren zugelassen.

Beide haben eine sehr gute und vergleichbare Wirksamkeit mit erster Besserung innerhalb von 30 min, ohne dass es zu einem sogenannten Rebound-Effekt, ein Wiederaufflackern der Schwellung, kommt. Zudem sind beide gut verträglich und nebenwirkungsarm. Äußerst selten sind anaphylaktische Reaktionen oder Thrombosen berichtet worden. Bei Abnahme der Effektivität nach längerer Anwendung sollte im Serum nach C1-INH-Antikörpern gefahndet werden [20]. Beide Produkte sind bei Motivation und Compliance der Betroffenen nach einer entsprechenden Schulung durch die behandelnden Ärzt:innen oder Diplom-Pflegekraft zur Selbstapplikation zugelassen. Allerdings können dies die Patient:innen aufgrund schlechter Venensituation oder bei Schwellung der Hände oder Arme in der Praxis häufig nicht selbst durchführen und müssen zu:r Ärzt:in. Vollständig implantierte Systeme (Portsysteme) oder Kathetersysteme, die über die Haut ausgeführt werden, können hilfreich sein.

##### 3.1.2. Icatibant

Dieser Bradykinin-2-Rezeptor-Antagonist dockt mittels kompetitiver Bindung an seinen Rezeptor in den Gefäßwänden an und dichtet diese somit ab, sodass die Bradykinin-Wirkung antagonisiert wird. Icatibant wurde von der Firma Jerini in Berlin, Deutschland entwickelt und unter dem Namen Firazyr® 2008 zur Zulassung zur subkutanen (s.c.) Behandlung von HAE-Attacken bei Erwachsenen gebracht. Jetzt wird es von Takeda, Tokio, Japan weltweit vertrieben. Die Wirksamkeit konnte in 3 umfangreichen multizentrischen RCT-Studien im Vergleich zu Placebo oder Tranexamsäure bei allen HAE-Attacken belegt werden [21, 22]. Bei etwa 10 % der behandelten HAE-Attacken kam es etwa 6 h nach der Injektion zu einem Wiederaufflackern der Schwellungen (Rebound-Phänomen), und eine zweite, sehr selten eine dritte Icatibant-Injektion waren erforderlich. Somit ist die maximale Dosis von Icatibant 30 mg s.c., 3 Spritzen innerhalb von 24 h. Zwischenzeitig ist das Präparat auch für Kinder ab 2 Jahren zugelassen, die Dosierung erfolgt gewichtsadaptiert und ist im Beipackzettel zu finden. Je früher zu Beginn einer Attacke Icatibant eingesetzt wird, desto schneller wirksam ist es [23]. Das Nebenwirkungsprofil ist sehr vorteilhaft, es kommt lediglich zu gering brennenden Schmerzen beim Injizieren sowie lokaler Rötung und Quaddelbildung, die sich nach kurzer Zeit wieder zurückbilden. Gelegentlich sind auch Schwächegefühl und Müdigkeit beschrieben, welche auch generell nach HAE-Attacken beobachtet werden.

In Österreich erhielten inzwischen mehrere Generika mit dem Wirkstoff Icatibant die Zulassung.

##### 3.1.3. Conestat alfa

Ein rekombinanter, humaner (rh) C1-INH (Ruconest®, Pharming, Leiden, Niederlande) ist seit 2011 zunächst nur für Erwachsene, rezent auch für Kinder und Jugendliche zugelassen zur Behandlung akuter Attacken bei HAE. Ruconest® wird durch eine rekombinante DNA-Technologie in den Milchdrüsen transgener Kaninchen produziert. In 3 RCT-Studien und einer Open-Label-Extension-Studie (OLE) zeigte es sich als hoch wirksam [24].

Seit 2017 ist es auch für die Selbstadministration zu Hause zugelassen. Die Dosierung erfolgt nach Körpergewicht (KG), bei Patient:innen bis zu 84 kg mit 50 Einheiten pro kgKG. Bei höherem KG werden 4200 Einheiten (2 Ampullen, in je 14 ml Lösungsmittel aufgelöst) intravenös appliziert.

Die häufigste Nebenwirkung des Präparates ist Kopfschmerz. Weil ein gesunder freiwilliger Proband mit Kaninchenallergie eine anaphylaktische Reaktion nach Gabe von rh-C1-INH entwickelte, entschied die European Medicines Agency (EMA), dass Ruconest® bei Patient:innen mit einer bekannten oder vermuteten Kaninchenallergie oder mit positivem IgE gegen Kaninchenallergene aufgrund des Risikos allergischer Reaktionen kontraindiziert ist.

##### 3.1.4. Gefrorenes Frischplasma

Gefrorenes Frischplasma, besser bekannt mit der englischen Bezeichnung Fresh-frozen Plasma (FFP), enthält C1-INH-Protein von gesunden Spender:innen und sollte daher bei akuten HAE-Attacken wirksam sein.

Außer einigen Fallberichten, die die Wirksamkeit von 500 ml FFP gezeigt haben, gibt es keine kontrollierten Studien. FFP ist nicht virusinaktiviert, nicht standardisiert und beinhaltet neben C1-INH-Protein auch andere Proteine aus dem Kallikrein-Kinin-System, die potenziell auch zur vermehrten Bradykinin-Bildung und Verschlechterung der akuten Attacke führen könnten [25]. Daher ist die Applikation von FFP bei HAE-Attacken nur Notsituationen vorbehalten, wo keine der zugelassenen Medikamente verfügbar sind.

#### 3.2. Unwirksame Therapeutika

Kortikosteroide, Antihistaminika, Adrenalin und seine Derivate, die bei Histamin-mediierten Angioödemen hochwirksam sind, führen bei HAE-Attacken (Bradykinin-mediierte Angioödeme) zu keinerlei Verbesserung und sollten bei bekannten HAE-Patient:innen erst gar nicht versucht werden.

#### 3.3. Welches Medikament für akute Attacken?

In Österreich stehen derzeit mehrere Medikamente für die Behandlung von akuten Attacken im Erwachsenenalter zur Verfügung: C1-INH-Konzentrate wie Berinert® und Cinryze®, Icatibant als Firazyr® bzw. mehrere Icatibant-Generika sowie rh C1-INH-Konzentrat als Ruconest®.

Alle sind hochwirksam. Es gibt jedoch keine Vergleichsstudien zwischen den einzelnen Medikamenten.

#### 3.4. Akuttherapie in der Schwangerschaft und Stillzeit

Beide aus humanem Plasma gewonnenen C1-INH-Konzentrate, Berinert® und Cinryze®, sind zur Behandlung akuter Attacken zugelassen.

Für die Behandlung mit Firazyr® gibt es zahlreiche Fallberichte [26], die über die Wirksamkeit und Verträglichkeit des Präparates berichten, jedoch keine größeren Fallzahlen oder Studien. Daher wird es in den Guidelines in der Schwangerschaft bisher nicht dezidiert empfohlen [1, 2]. Die Anwendung im Bedarfsfall kann aber auch nicht als Kunstfehler angesehen werden. Für die Stillperiode kann für Firazyr® auch noch keine Empfehlung abgegeben werden, da es nicht untersucht wurde, ob es in die menschliche Muttermilch übergeht.

Zu Ruconest® liegen bisher zu wenig Erfahrungen bei Schwangeren und stillenden Frauen vor. Die Anwendung während der Schwangerschaft oder Stillzeit wird daher in den bestehenden Leitlinien nicht empfohlen.

#### 3.5. Akuttherapie im Kindesalter

Für die medikamentöse Behandlung von akuten HAE-Attacken im Kindesalter ist Berinert® in der gleichen gewichtsadaptierten Dosierung wie bei Erwachsenen zugelassen. Cinryze® ist ab 2 Jahren zugelassen, Kinder zwischen 2 und 11 Jahren und einem KG zwischen 10 und 25 kg erhalten eine Dosis von 500 IE, bei einem KG von über 25 kg 1000 IE intravenös. Seit 2017 ist auch Firazyr® für Kinder und Jugendliche von 2 bis 17 Jahren zur subkutanen Therapie von akuten Attacken gewichtsadaptiert zwischen 1 und 3 ml zugelassen. In einer unkontrollierten Studie mit 32 Patient:innen im Alter von 2 bis 17 Jahren zeigte sich bei mehr als 90 % der Patienten eine deutliche Besserung der Schwellung 2 h nach Injektion [27].

Ruconest® ist für Jugendliche (ab 12 Jahren) zugelassen. Über die Sicherheit und Wirksamkeit von Ruconest® bei Kindern im Alter von 0 bis 12 Jahren gibt es noch keine Daten.

##### Empfehlung 10

Wir empfehlen, dass alle Attacken für eine On-demand-Behandlung in Betracht gezogen werden. Jede Attacke, welche die oberen Atemwege betrifft oder betreffen könnte, soll behandelt werden. Attacken sollen so früh wie möglich behandelt werden.

##### Empfehlung 11

Wir empfehlen, dass alle Patienten über ausreichende Medikamente für die Notfallbehandlung von zumindest 2 Attacken verfügen und diese Bedarfsmedikation immer bei sich tragen.

#### 3.6. Medikamentöse Langzeitprophylaxe

Alle HAE-Patient:innen sollten zunächst eine Bedarfstherapie für akute HAE-Attacken erhalten. Wenn sich damit keine hinreichende Beschwerdenkontrolle erreichen lässt, sollte eine Langzeitprophylaxe in Erwägung gezogen werden.

Für die LTP ist neben der Effektivität der Präparate die Verträglichkeit ein wichtiger Aspekt, da sie über Jahre bis Jahrzehnte eingesetzt werden.

##### 3.6.1. C1-INH-Konzentrat

Cinryze® (Takeda, Japan) reduzierte in einer RC-Cross-over-Studie (22 Patient:innen in zwei 12-Wochen-Perioden) die HAE-Attackenzahl von 12,7 auf 6,3 mit deutlicher Reduktion der Schwere und Dauer der verbliebenen Attacken [28]. Das erlaubte eine Zulassung in der Dosierung 1000 IE 2‑mal wöchentlich i.v. für die LTP 2008 in den USA und 2011 in Europa. Ab 2017 kam auch die EU-Zulassung für LTP für Kinder – ab dem sechsten Lebensjahr.

Berinert® 2000/3000 (CSL Behring, Marburg, Deutschland), ein konzentriertes, Volumen-reduziertes C1-INH-Konzentrat aus humanem Plasma, wurde für die LTP von HAE-Attacken zur s.c.-Applikation, 2‑mal/Woche, entwickelt [29]. Die empfohlene Dosierung beträgt 60 U/kg KG. Das Präparat hat sich in klinischen Studien als sicher und wirksam erwiesen. Dank der besseren Symptomkontrolle kam es zugleich zu verbesserter Lebensqualität bei Patienten mit relativ häufigen HAE-Attacken im Vergleich zur On-demand Therapie [30]. Die s.c.-Applikation ist an sich unproblematisch, kann jedoch wegen der relativ großen Menge zu lokaler Spannung, Schmerzen, Schwellung, Blutergüssen und Juckreiz führen. Die seltenen thromboembolischen Ereignisse dürften bei Vorliegen von zusätzlichen Risikofaktoren aufgetreten sein [31].

Die Zulassung erfolgte in Österreich 2020 für Kinder ab 6 Jahren, Jugendliche und Erwachsene [32].

##### 3.6.2. Kallikrein-Inhibitoren

Lanadelumab (Takhzyro®, Takeda, Tokio, Japan) ist ein subkutan injizierbarer, vollständig humaner, monoklonaler Antikörper gegen Plasma-Kallikrein und hat sich in klinischen Studien als sehr wirksam in der LTP von HAE-Attacken erwiesen [33]. Es ist in Österreich seit 2019 als erstes Biologikum zur LTP von HAE bei Erwachsenen und Kindern ab 12 Jahren zugelassen und als 300 mg Fertigspritze erhältlich. Es verhindert HAE-Attacken durch Inhibition von Plasma-Kallikrein. Als LTP wird es typischerweise alle 2 Wochen s.c. verabreicht, ein konstanter Plasmaspiegel wird nach etwa 7 Wochen erreicht. Nach 6 Monaten kann bei kompletter HAE-Attacken-Freiheit ein Dosisintervall von 300 mg alle 4 Wochen in Betracht gezogen werden. Nebenwirkungen sind Schmerzen sowie ein Erythem an den Injektionsstellen, gelegentlich Kopfschmerzen, Schwindelgefühl, Ausschlag, Myalgien, Arthralgien, Anstieg der Lebertransaminasen, insgesamt waren die berichteten Nebenwirkungen jedoch nicht wesentlich häufiger als in der Placebogruppe. Die Kinderstudie in der Altersklasse 2 bis 11 Jahre ist bereits positiv abgeschlossen, und die Zulassung konnte 2023 erfolgen [34].

Berotralstat (Orladeyo®, BioCryst, Durham, NC, USA) ist ein Kallikrein-Inhibitor, der die proteolytische Aktivität von Kallikrein hemmt. Es ist ein eigens für LTP von HAE-Attacken entwickeltes „small molecule“ mit dem Vorteil, dass es im Gegensatz zu Biologika oral verabreicht werden kann [35].

Es wird typischerweise in einer Dosis von 150 mg 1‑mal täglich oral zu oder nach einer Mahlzeit eingenommen und wurde 2021 in Österreich zugelassen. In 3 klinischen Studien zeigte sich Berotralstat als sicher und effektiv in der LTP von HAE-Attacken [36]. Die häufigsten Nebenwirkungen sind Gastritis, Bauchschmerzen, Erbrechen und Durchfall, gelegentlich Kopfschmerzen, wobei sie meist zu Beginn der Therapie auftreten und mit der Zeit sistieren. Da es über CYP450 abgebaut wird, ist Vorsicht bei lang andauernder Kombination mit anderen Medikamenten geboten, die auch über dasselbe Leberenzym metabolisiert werden [36]. Aktuell werden für eine Phase-3-Studie Kinder zwischen 2 und 11 Jahren rekrutiert.

Zusammengefasst, können alle bisher genannten Medikamente als First-line-Therapie für die LTP von HAE-Attacken gleichermaßen empfohlen werden. Daher wird die Entscheidung je nach Lebensstil, Probleme mit Spritzen, beruflichem und familiärem Engagement (Zeitmanagement) im Einzelfall gemeinsam mit dem Betroffenen gefällt („shared decision-making“) [37].

##### Empfehlung 12

Wir empfehlen, dass Patient:innen bei jedem Besuch auf die Indikation für eine Langzeitprophylaxe beurteilt werden. Krankheitsaktivität, -belastung und -kontrolle wie auch die Patientenpräferenz sollten dabei berücksichtigt werden (Tab. 4).

**Table 4:** 

Akuttherapie	Langzeitprophylaxe
*pd C1-INH* (Berinert®)	*Berotralstat *(Orladeyo®)
*pd C1-INH* (Cinryze®)	*C1-INH* (Cinryze®)
*rh C1-INH* (Ruconest®)	*C1-INH* (Berinert® 2000/3000)
*Icatibant* (Firazyr®, Icatibant-Ratiopharm®, Icatibant ACC®)	*Lanadelumab *(Takhzyro®)

##### 3.6.3. Attenuierte Androgene

Ab Mitte der 1970er-Jahre, in einer Ära, in der keine wirksamen Therapeutika zur Verfügung standen, wurden abgeschwächte Androgenderivate wie Danazol für eine Langzeitprophylaxe von HAE eingesetzt. In einer Studie zeigte sich bei 46 % der Betroffenen eine Wirksamkeit in der Attackenreduktion [38]. Dies erfolgt durch Anhebung des C1-INH-Spiegels im Serum. Danazol-hältige Medikamente sind mit massiven kurzfristigen Nebenwirkungen vor allem bei Frauen (Gewichtszunahme, Menstruationsstörungen bis zur Amenorrhö und Virilisierung) [39] sowie bei beiden Geschlechtern längerfristig mit Hepatotoxizität, Depression, arterieller Hypertonie und hämorrhagischer Zystitis, Leberzelladenomen und -karzinomen [40] behaftet.

Aus diesen Gründen und angesichts besser wirksamer und verträglicher Medikamente werden Androgene in Österreich seit mehreren Jahren von Experten nicht mehr zur LTP empfohlen [41]. Diese Medikation sollte sofort umgestellt werden, wenn ein Patient noch damit in Behandlung ist. Die Listung als Zweitlinientherapie in den internationalen Leitlinien [2] liegt an der Tatsache, dass in vielen Ländern der Erde die Mittel der ersten Wahl nicht zur Verfügung stehen!

##### 3.6.4. Tranexamsäure

Ab 1972 wurden Tranexamsäure und ähnliche Antifibrinolytika zur Langzeitbehandlung von HAE-Attacken eingesetzt [42]. Die Wirksamkeit der Antifibrinolytika ist bei Erwachsenen deutlich geringer als die von attenuierten Androgenen bzw. den neueren Substanzklassen. Kontraindikationen sind Schwangerschaft, Niereninsuffizienz, akute Thrombose oder thromboembolisches Geschehen in der Eigen- und/oder Familienanamnese. Mögliche Nebenwirkungen sind gastrointestinale Beschwerden, Myalgien mit Kreatininkinaseerhöhung und Sistieren der Menses bei Frauen.

Auch diese Pharmaka sollten angesichts besser wirksamer und verträglicher Medikamente am österreichischen Markt nicht mehr für die Indikation HAE verwendet werden.

##### 3.6.5. Gestagene

Gestagene können bei der LTP von HAE-Patientinnen hilfreich sein. Eine Zulassung oder RCT-Studien hierzu gibt es nicht, jedoch Berichte über Therapieerfolge in Fallserien [43]. Desogestrel ist eine „Progesteron-only-Pille“ (POP) und bei HAE-Patientinnen das Antikonzeptivum der Wahl, da sich die Erkrankung bei Östrogeneinnahme verschlechtert. Etwa zwei Drittel der Frauen berichten unter Gestagen über eine Symptomverbesserung. Eine POP sollte jedoch niemals vorrangig als LTP-Therapie eingesetzt werden, sondern lediglich als adjuvante Maßnahme, wenn orale Kontrazeptiva verwendet werden.

#### 3.7. Medikamentöse Kurzzeitprophylaxe

HAE-Patient:innen sollten 1 h vor allen Interventionen, wo es zu mechanischer Manipulation der oberen Luft- und Speisewege – wie zahnärztliche Operation, Zahnextraktion, andere Operationen im Mund-Rachen-Bereich, Intubation – kommt, ein C1-INH-Konzentrat zur Kurzzeitprophylaxe erhalten [44].

Für Berinert® wird vom Hersteller die Gabe von 1000 IE für Erwachsene und 15–30 IE/kg KG für Kinder und Jugendliche innerhalb von 6 h vor dem Eingriff und für Cinryze® vom Hersteller eine Kurzzeitprophylaxe mit 1000 IE innerhalb von 24 h vor einem Eingriff empfohlen.

Trotz der Kurzzeitprophylaxe können nicht alle Attacken vermieden werden, sodass in den nachfolgenden 24 h weitere Medikation zur Behandlung einer später auftretenden HAE-Attacke bereitstehen sollte [44].

##### Empfehlung 13

Wir empfehlen, eine kurzfristige Prophylaxe vor medizinischen, chirurgischen oder zahnärztlichen Eingriffen sowie vor anderen Angioödem-auslösenden Ereignissen zu erwägen.

#### 3.8. Zukünftige Entwicklungen

Die derzeit geprüften neuen Therapieoptionen haben einerseits das Ziel, mit weniger Verabreichungen den Betroffenen ein normales Leben zu ermöglichen oder gar mit einer einzigen Verabreichung sie zu heilen, andererseits wird aufgrund der vorteilhaften oralen Einnahme an der Erweiterung der oral verabreichten Wirkstoffe sowohl für prophylaktische als auch für On-demand-Anwendung gearbeitet.

##### 3.8.1. Orale Medikamente in Entwicklung

Sebetralstat (KVD 900) ist ein „small molecule“ für akute Attackenbehandlung (on-demand) und zielt auf Plasma-Kallikrein-Hemmung. In einer Phase-2-Studie erreichte 600 mg Sebetralstat eine rasche Symptombesserung und Rückgang der HAE-Attacken [45]. Aktuell läuft die Sebetralstat-Phase-3-Studie für Kinder ab 12 Jahren und Erwachsene.

PHA 022121 (PHSV416) ist ebenfalls ein „small molecule“, welches auf die Hemmung des Bradykinin-B2-Rezeptors zielt. Es ist in Evaluation sowohl für die On-demand-Therapie als auch für eine LTP.

Eine Phase-2-Studie zur Evaluation der Wirksamkeit von PHA 022121 für die On-demand-Therapie wurde gerade abgeschlossen. Alle hierbei getesteten Konzentrationen (10, 20 und 30 mg) reduzierten die Schwellungen 4 h nach Einnahme im Vergleich zu Placebo hochsignifikant [46]. PHA 022121 wird in einer abgeänderten Galenik auch für die LTP von HAE evaluiert. Die Phase-2-Studie hierzu ist aktiv, die Rekrutierung ist jedoch abgeschlossen.

##### 3.8.2. Parenterale Medikamente in Entwicklung

Garadacimab ist ein monoklonaler IgG4-Antikörper, gerichtet gegen aktivierten Faktor XII [47]. In der vor Kurzem publizierten Phase-3-Studie zeigte die subkutane Injektion von 200 mg 1‑mal pro Monat eine durchschnittliche HAE-Attacken-Reduktion um 87 % im Vergleich zu Placebo [48]. Die Zulassung und Markteinführung sollen demnächst folgen.

Donidalorsen ist ein Antisense-Oligonukleotid aus 24 Nukleotiden, konjugiert an ein N‑Acetylgalactosamin, welches seine Aufnahme in die Hepatozyten erleichtert. Es hemmt die Plasma-Kallikrein-Produktion durch Degradierung der Präkallikrein-mRNAs. In einer Phase-2-Studie wurde die subkutane Applikation von 80 mg Donidalorsen 1‑mal pro Monat über 4 Monate bei 20 Patienten evaluiert (14 bekamen Donidalorsen und 6 Placebo) [49]. Patient:innen, die in der Verum-Gruppe waren, hatten eine durchschnittliche HAE-Attacken-Reduktion von 90 % im Vergleich zu Placebo. Nach der zweiten Applikation erreichte die Reduktion sogar 97 %.

Es kam zu keinen nennenswerten Nebenwirkungen, die AE-QoL verbesserte sich enorm. Phase-3- und Open-label-extension-Studien sind derzeit aktiv mit 80 mg Donidalorsen alle 4 oder alle 8 Wochen.

##### 3.8.3. Entwicklungen zur Gentherapie,

die durch Eingriff in diversen pathogenetisch relevanten Schritten die Heilung der Betroffenen ermöglichen sollten.

NTLA-2002 basiert auf mRNA- und CRISPR-Cas9-(Cas9-Endonuklease-System)-Technologie und hemmt die Präkallikrein-Produktion in Hepatozyten.

Zurzeit läuft die klinische Phase-1/2-Studie, Zwischenanalysen zeigten eine durchschnittliche Reduzierung der HAE-Attacken um 94 % bzw. 90 % nach einer Einzelinfusion von 25 bzw. 75 mg NTLA-2002 über eine 6‑ bis 9‑Monate-Nachbeobachtungsphase. Hierbei kam es zu einer dosisabhängigen, anhaltenden mittleren Reduzierung des Kallikreins um 64 % bzw. 92 %. Obwohl NTLA-2002 von bisherigen Studienpatienten gut vertragen wurde, sind Langzeitsicherheitsdaten noch ausständig. Bisher gibt es noch keine Veröffentlichungen, sondern nur Kongressberichte [50].

BMN 331 ist eine in der Entwicklung befindliche, Adeno-assoziiertes Virus 5 (AAV5)-basierte Gentherapie, welche auf die Korrektur des Gendefekts auf dem *SERPING1*-Gen abzielt. Bisher wurden keine klinischen Daten aus einer Phase 1/2 zur Bewertung der Sicherheit und Wirksamkeit von BMN 331 veröffentlicht, wobei diese AAV5-basierte Gentherapie bereits bei Hämophilie A erfolgreich eingesetzt wurde. Potenzielle Risiken sind Immunogenität, da 50 % der Patienten durch Entwicklung von Antikörpern gegen AAV5-Therapie resistent wurden. Hepatotoxizität, der lange Einsatz von Kortikosteroiden und Genotoxizität stellen weitere potentielle Gefahren dar [51].

Es bleibt abzuwarten, ob sich diese Therapieformen durchsetzen werden.

## XI. Prognose

Die Mehrzahl der Patient:innen mit HAE wird lebenslang mit dem Auftreten von Angioödemattacken rechnen müssen, durch die neu verfügbaren Therapieoptionen kann die Frequenz der Attacken jedoch drastisch reduziert werden.

Die für behandelnde Ärzt:innen und naturgemäß noch mehr die betroffenen Patient:innen drängendste prognostische Frage ist, mit welcher Wahrscheinlichkeit beim jeweiligen Patienten mit einem potenziell lebensbedrohlichen Larynxödem zu rechnen ist. Obwohl solche Schwellungen nur in ca. 1 % der Attacken auftreten, muss immerhin etwa die Hälfte aller Patient:innen zumindest 1‑mal im Leben mit einem derartigen Ödem rechnen [52]. Vor Verfügbarkeit einer spezifischen Therapie endeten ca. 30 % solcher Attacken tödlich [53]. Selbst nach Einführung neuerer Therapieformen werden immer wieder tödliche Larynxödeme berichtet [8]. Dabei sind vorwiegend Patienten im Alter zwischen 11 und 45 Jahren von solchen gefährlichen Attacken betroffen [54]. Kinder mit einem derartigen Larynxödem stellen nicht nur diagnostisch ein Problem dar (auch hier ist das ungenügende Ansprechen auf die zur Behandlung histaminbedingter Ödeme üblichen Therapeutika Antihistamine, Kortikoide und auch Epinephrin der wegweisende klinische Hinweis), aufgrund des geringeren Durchmessers der Atemwege tritt die Gefährdung auch rascher ein [55]. Klinische Symptome eines Larynxödems sind Globusgefühl, Dysphagie, Stimmveränderungen und Heiserkeit sowie Atemnot, manchmal sieht man auch begleitende Schwellungen des weichen Gaumens, der Uvula und der Zunge, diese Schwellungen können jedoch auch unabhängig vom Larynxödem auftreten. Von den ersten Symptomen eines Larynxödems bis zur maximalen, lebensbedrohlichen Manifestation dauert es meist zwischen 8 und 12 h, dies kann jedoch im Einzelfall auch viel schneller verlaufen [54]. Die überwiegende Mehrheit der Ödeme (97,4 %) betrifft die Haut bzw. gastrointestinale Attacken [54].

Nachdem die meisten Todesfälle bei nicht diagnostizierten Patienten auftreten [8], kommt einer möglichst raschen Diagnostik mit Einleitung einer adäquaten Therapie große Bedeutung zu. Hier ist es in den letzten Jahrzehnten glücklicherweise zu einer deutlichen Verkürzung des Intervalls von der Erstmanifestation bis zur Diagnose gekommen, nicht zuletzt durch Erhöhung der Awareness bei Patienten und Medizinern.

Nicht unerwähnt soll bleiben, dass nicht bei allen Personen, die vom Gendefekt betroffen sind, die Krankheit jemals manifest wird. Festgehalten werden muss aber auch, dass sich die Krankheit erstmals schon in den ersten Lebenstagen manifestieren kann, selten dies aber erst jenseits des 60. Lebensjahres erstmals tut – dann muss ein erworbener C1-INH-Mangel ausgeschlossen werden.

## XII. Zusammenfassung

Die Einstufung des hereditären Angioödems als „orphan disease“ hat der Beforschung der Erkrankung und der Entwicklung von Behandlungsmethoden großen Schwung verliehen. Pharmazeutische Fortschritte, die zur Zulassung von sicheren und gut wirksamen Medikamenten für die Behandlung von Attacken ebenso wie zur Prävention ebensolcher geführt haben, gemeinsam mit der Schulung Betroffener zur frühen Selbstmedikation und deren umfassende Aufklärung sollten heute jedem:r HAE-Patient:in ein weitgehend normalisiertes Leben ermöglichen. Voraussetzung dafür sind aber ein individualisierter Aktionsplan und die Betreuung in einem spezialisierten Zentrum – zu heterogen und komplex sind die krankheitsbedingten Probleme bei dieser seltenen Erkrankung für den einzelnen Betroffenen. Historische Produkte wie die attenuierten Androgene, Antifibrinolytika oder Fresh-frozen-Plasma sollten heute nicht mehr eingesetzt werden, da bei Ersteren das Nebenwirkungspotenzial zu hoch bzw. den anderen die Wirksamkeit nicht dokumentiert ist – auch wenn diese in der internationalen Leitlinie noch als Mittel der zweiten Wahl geführt sind. Die Selbstmedikation mit Mitteln der ersten Wahl sollte HAE-Patient:innen das Behandlungsziel ermöglichen, ein anfallsfreies, unbeschwertes Leben ohne Angst vor und der Gefahr von potenziell lebensbedrohlichen Beeinträchtigungen zu führen.

## Abkürzungen

C1-INH: C1-Esterase-Inhibitor
EMA: European Medicines Agency
FFP: Fresh-frozen plasma
HAE: Hereditäres Angioödem
HAE-C1-INH: HAE, bedingt durch C1-INH-Mangel
HAE-nC1-INH: HAE mit normalem C1-INH-Spiegel
i.v.: Intravenös
LTP: Langzeitprophylaxe
KG: Körpergewicht
OLE: Open-Label-Extension-Studie
RCT: Randomisierte, kontrollierte Studie
s.c.: Subkutan

## Interessenkonflikt

W. Aberer ist oder war kürzlich Sprecher/Advisor oder Studienarzt für BioCryst, CSL Behring und Takeda. S. Altrichter ist oder war kürzlich Sprecherin/Advisorin oder Studienärztin für AstraZeneca, BioCryst, CSL Behring, LEO Pharma, Moxie GmbH, Novartis, Pharvaris, Sanofi, Takeda, ThermoFisher. U. Cerpes ist oder war kürzlich Sprecher/Advisor oder Studienarzt für AbbVie, Almirall, Galderma, Kiniksa Pharmaceuticals, Leo Pharma, Lilly, Menlo Therapeutics, Mylan, Novartis, Pfizer, Sanofi, Takeda. T. Hawranek ist oder war kürzlich Sprecher/Advisor für Takeda. C. Schöffl ist oder war kürzlich Sprecher/Advisor oder Studienarzt für BioCryst, CSL Behring, KalVista, Novartis und Takeda. T. Kinaciyan ist oder war kürzlich Sprecherin/Advisorin oder Studienärztin für BioCryst, CSL Behring, KalVista, Kiniksa, Novartis, Pharvaris, Sanofi und Takeda.
